# Bioactive compounds as potential alternative treatments to prevent cancer therapy-induced male infertility

**DOI:** 10.3389/fendo.2023.1293780

**Published:** 2024-01-18

**Authors:** Layla Simón, María Salomé Mariotti-Celis

**Affiliations:** Nutrition and Dietetic School, Facultad de Medicina, Universidad Finis Terrae, Santiago, Chile

**Keywords:** fertility, testicular, chemotherapy, radiation, tumor, resveratrol, curcumin

## Abstract

About 8-12% of couples experience infertility, with male infertility being the cause in 50% of cases. Several congenital and acquired conditions, including chronic diseases and their treatments, can contribute to male infertility. Prostate cancer incidence increases annually by roughly 3%, leading to an increment in cancer treatments that have adverse effects on male fertility. To preserve male fertility post-cancer survival, conventional cancer treatments use sperm cryopreservation and hormone stimulation. However, these techniques are invasive, expensive, and unsuitable in prepubertal patients lacking mature sperm cells. Alternatively, nutritional therapies enriched with bioactive compounds are highlighted as non-invasive approaches to prevent male infertility that are easily implementable and cost-effective. In fact, curcumin and resveratrol are two examples of bioactive compounds with chemo-preventive effects at the testicular level. In this article, we summarize and discuss the literature regarding bioactive compounds and their mechanisms in preventing cancer treatment-induced male infertility. This information may lead to novel opportunities for future interventions.

## 1 Introduction

Infertility is a major health problem that affects approximately 8% to 12% of couples worldwide. Male infertility stands as the primary cause in 20% to 30% of cases and contributes to infertility in an additional 20% of couples. Collectively, male factors account for 50% of infertility cases. Various congenital, acquired and idiopathic factors contribute to male infertility. In terms of acquired factors, chronic diseases and their treatments, such as cancer, chemotherapy and radiation, respectively, play a role in male infertility (1).

Cancer is a major health problem worldwide, with incidence and death rates historically higher in men than women. In fact, the probability of developing invasive cancer within lifetime was 1 in 2 men compared to 1 in 3 women in the United States between 2017-2019. Indeed, men are more exposed to carcinogenic factors, such as endogenous hormones, smoking, height, and immune response. In this sense, it is expected that around one-half of cancers in men will be concentrated in prostate (29%), lung (12%) and colorectal (8%) cancers in 2023. In the United States, prostate cancer incidence has increased by roughly 3% annually, similar to lung cancer (2%) (2). Therefore, the sustaining increase in cancer incidence leads to an increment in cancer treatments that can have adverse effects on male fertility.

Hopefully, the 5-year cancer survival rate has increased from 49% in the mid-1970s to 68% in the last decade. Moreover, prostate cancer (97%) has the highest survival rate after thyroid cancer (98%) (2), thereby increasing the number of patients surviving after cancer treatment.

Male infertility after cancer treatment is caused by: (a) decreased gonadotropin secretion from the pituitary gland caused by immune inhibitors, cranial irradiation and central nervous system tumors surgeries; (b) spermatogenic dysfunction due to chemotherapy or irradiation; (c) obstruction of seminal tracts caused by intrapelvic surgeries; (d) sexual dysfunction due to intrapelvic or retroperitoneal surgeries or irradiation. In fact, almost 46% of young cancer survivors overcome male infertility, and 30% of patients have testicular dysfunction due to chemo- or radio-therapy (3). Unfortunately, male survivors of childhood cancer have a higher desire for children compared with their siblings (25% *vs*. 7%) (4), thereby developing interventions that preserve male fertility is a necessity for cancer patients.

Adult cancer patients use sperm cryopreservation and hormone stimulation to preserve male fertility post-cancer treatments. However, these techniques are invasive, expensive, and unsuitable in prepubertal patients lacking mature sperm cells. Using less gonadotoxic chemo- and radio-therapies, organ-sparing surgeries and cryopreserving testicular tissue are some methods to preserve or restore fertility in prepuberal males undergoing cancer therapies. Unfortunately, these approaches are even more expensive, invasive, possible only for some patients and available in only few medical centers (5).

On the other hand, nutritional therapies enriched with bioactive compounds seem to be cost-effective, easily-implementable, and non-invasive approaches to prevent male infertility. In a metanalysis review, L-Carnitine administrated with micronutrients, antioxidants and herbal supplements increases pregnancy rates (6). In fact, antioxidants such as L-Carnitine, Coenzyme-Q10, ω3 fatty acid and selenium improves sperm quality parameters (7).

In this article, we summarize and discuss the literature concerning bioactive compounds and their mechanisms of action within preventing cancer treatment-induced male infertility. This information may contribute to develop novel opportunities for future interventions.

## 2 Etiology of male infertility

Male infertility is classified into four categories: (a) hypothalamic-pituitary axis disturbances, (b) spermatogenic qualitative and (c) quantitative defects, and (d) ductal obstruction or dysfunction (8). Independently of the category, about 30% of infertility cases are due to idiopathic conditions, and 70% are caused by genetic mutations or acquired conditions (9).

Genetic mutations affect almost 15% of males with infertility and 25% of men with azoospermia (no spermatozoa in the ejaculate). Some genetic alterations are chromosomal numerical or structural abnormalities, Y chromosomal deletions, azoospermia factor (AZF) deletions, androgen receptor (AR) gene mutations, cystic fibrosis transmembrane conductance regulator (CFTR) gene mutations (8).

Some acquired conditions that affect male fertility are obesity, pesticide exposure, smoking and medications (9). For instance, obesity is a health problem that course with concomitant diseases such as cardiovascular disease, type 2 diabetes and cancer. Moreover, obesity affects the hypothalamic-pituitary-gonadal axis, disrupts testicular steroidogenesis, and induces erectile dysfunction, poor semen quality and prostatitis. Some mediators of infertility-induced obesity are hyperinsulinemia, hyperleptinemia, chronic inflammation and oxidative stress (10). In the case of pesticide exposure, workers and exposed populations have deleterious semen quality (volume and sperm count, motility and morphology), DNA fragmentation and chromosome aneuploidy (11). Males with smoking habits have a high probability of being infertile, which increases with long smoking duration (>10 years) (12). Also, medications have adverse effects on reproductive achievement. For example, males receiving major antidepressant drugs have reduced conceptional rates (13).

Similarly, other environmental pollutants that have shown to affect male fertility are heavy metals, microplastics and endocrine-disrupting chemicals such as bisphenols, phthalates, and parabens. Heavy metals such as Zn, Se, Pb, and Cd are known to increase lipid peroxidation, reduce antioxidant capacity, and thereby impair sperm function (14). Also, high blood and seminal levels of Pb, Cd, Ba and U are associated with low sperm viability (15). In the case of microplastics, it has been proved in animals that microplastics impair semen quality in an equivalent human dose of 0.016 mg/kg/d, which is nearly achieved in Japan and South Korea (16). In addition, bisphenols, phthalates, and parabens increase ROS production, DNA damage and apoptosis, leading to abnormal sperm count and semen quality (17).

Testicular tumors represent almost 2% of all cancers in men. Testicular tumors are classified into two main groups: germ cell and sex cord tumors. Germ cell tumors include germ cell neoplasia *in situ*, seminoma, teratoma, non-seminomatous germ cell tumors, embryonal carcinoma, Yolk sac tumors, trophoblastic and regressed germ cell tumors. Sex cord stromal tumors cover Sertoli and Leydig cells tumors, and myoid gonadal stromal tumors (18). Although testicular cancer is a disease that reduces male fertility by disturbing spermatogenesis, vasculogenesis and the secretion of paracrine factors, the reduction in fertility is more a consequence of cancer treatment than the primary testicular tumor effects. In the case of surgery, 50% of patients after orchiectomy have a decrease in sperm number. Radiotherapy effects on spermatogenesis depend on doses. In fact, doses less than 1 Gy allow spermatogenesis recovery after 18 months, doses up to 3 Gy after 30 months, and up to 4 Gy after 5 years. Moreover, the chemo- and radiotherapy combination increases their gonadotoxic effects that when are administrated alone. The main adverse effect of chemotherapy on fertility is a consequence of targeting proliferating cells as germ cells (19). Spermatogonia are sensitive to the cytotoxic effects of cancer treatments, with increased susceptibility during the spermatogenesis undergoing differentiation (3). In addition, chemotherapy induces mutations in stem cell spermatogonia, thereby causing permanent damage to spermatogenesis. Some alkylating agents such as vincristine, prednisolone and cyclophosphamide induce permanent germ cell depletion and azoospermia in 80% of patients. Etoposide and doxorubicin promote azoospermia in 90% of patients (19).

In addition, other types of tumors dismiss male fertility by endocrine, nutritional, metabolic and immune alterations (19). In fact, germ cells are sensitive to the toxic effects of cancer therapy. Due to spermatogonia are more sensitive than highly differentiated germ cells, initially cancer treatment reduces the number of spermatogonia but does not affect the number of spermatocytes, spermatids and sperm cells. So, the sperm count is maintained at the beginning of the treatment but is reduced dramatically after 1 or 2 months. The azoospermia occurs after 12 weeks of cancer treatment, and in the case of low cytotoxic drugs (vinblastine, bleomycin, methotrexate, 5-fluorouracil), sperm count is recovered after 12 weeks of discontinuation chemotherapy. But, the highly cytotoxic drugs (cyclophosphamide, cisplatin, busulfan) induce more prolonged and even permanent azoospermia. Furthermore, cranial surgery or irradiation affect the hypothalamic-pituitary-gonadal axis, reduce gonadotrophin secretion and thereby spermatogenesis (3).

## 3 Treatment for male infertility

### 3.1 Conventional treatments preventing male infertility

Treatments for male fertility are selected in function of the etiology. For instance, male hypogonadotropic hypogonadism is secondary to gonadotropin deficiency and is treated with hormone stimulation to replace the missing hormones. Therapeutic GnRH stimulates pituitary gonadotropin secretion. Gonadotropins imitate LH and FSH. Selective Oestrogen Receptor Modulators (SERMs) inhibit Oestrogen Receptors in the hypothalamus and pituitary and suppress the oestrogen-mediated negative feedback on the hypothalamic-pituitary-gonadal axis. Aromatase inhibitors inhibit the conversion of testosterone to oestradiol, thereby decreasing the oestradiol-mediated negative feedback (20). Although hormone stimulation has promising effects on animals, it is ineffective in human cancer patients (5).

In the case of male infertility due to cancer treatment in reproductive age, an alternative to preserve fertility is sperm cryopreservation followed by *in vitro* fertilization or intracytoplasmic sperm injection. These approaches are effective when sperm is collected for 2-3 ejaculates and before the cancer treatment. But, in some cases starting cancer treatment is an urgency that no contemplate time for sperm cryopreservation. Moreover, this option is expensive and difficult to develop in some centers (19). Furthermore, sperm cryopreservation and assisted-reproductive technology have 30% pregnancy and 25% live birth rates, respectively. Although being compared to general fertility treatment, is not effective in all the patients (3).

Cryopreserving sperm is impossible in prepuberal patients. The cryopreservation of testicular tissue obtained through a biopsy is possible in these patients (19). Moreover, cryopreserving testicular tissue is the only option in men unable to ejaculate and those with azoospermia (3). However, testicular biopsy has potential risks in leukemia patients. Furthermore, fertility restoration is still a problem in young cancer survivors for clinical reasons (19). In fact, the alternatives for using cryopreserving spermatogonia stem cells are under experimentation. For instance, testicular tissue auto- or xeno-grafting, spermatogonial stem cells transplantation and *in vitro* spermatogenesis are being studied in animals (5). However, culturing sperm and germ cells has been unsuccessful in humans. In addition, these approaches have some disadvantages: the small number of spermatogonia stem cells, contamination with cancer cells, surgical procedures, intracytoplasmic sperm injection and virus infection (3).

### 3.2 Bioactive compounds as potential alternative treatments preventing male infertility

Regarding nutritional aspects, abnormal sperm parameters and hormone levels are associated with a high intake of alcohol, processed starchy and meat foods and foods rich in trans and saturated fatty acids (21–27). On the other hand, nutritional healthy habits such as high consumption of vegetables, fruits and seafood products have shown a positive association with the prevention of male infertility (28).

The Western diet is rich in saturated fatty acids, carbohydrates and proteins found in processed foods. It is also reduced in polyunsaturated fatty acids, dietary fibers and antioxidants, consequently having a negative effect on sperm quality. In this sense, the Western diet is associated with obesity, dyslipidemia, insulin resistance, oxidative stress, and aromatase activity. As a result, the Western diet reduces testosterone levels leading to a decrease in sperm count, motility and morphology. Moreover, the Western diet modifies the metabolism of sperm cells by decreasing glycolysis and mitochondrial respiration, reducing ATP content and sperm motility. Contrary, vegetarian diets are rich in plant-based antioxidants such as polyphenols with beneficial effects on sperm quality. For instance, quercetin is a flavonoid that interacts with mitochondrial membranes at the coenzyme Q-binding site, suppresses superoxide generation and promotes ATP synthesis (29). Similarly, the Mediterranean diet is characterized by a high intake of polyphenols, monounsaturated fatty acids, fiber, ω3 fatty acids, and vitamins, while being reduced in saturated and trans-fatty acids. Therefore, the Mediterranean diet is associated with a positive impact on male fertility, high sperm count and motility (28). Furthermore, the Mediterranean diet reduces oxidative stress and increases ATP generated through energetic metabolism, thus improving sperm quality (29).

As we previously described, there exists an intricate connection between nutritional habits and fertility. *In vivo* models have confirmed that administrating several bioactive compounds found in plant sources can help to mitigate the detrimental effects caused by unhealthy habits and environmental pollutants, thereby aiding in the recovery of fertility. **Table 1** summarizes the nutritional habits known to prevent male infertility, classifying the sources and bioactive compounds that control the detrimental effects.

**Table 1:** Bioactive compounds preventing male infertility.

Source or Bioactive Compound	Model	Effect	Reference
Olive oil	Rabbit model of high-fat diet-induced hypercholesterolemia	Recover the semen quality and sperm function	(30–32)
*Lepidium meyenii* (Maca)	Healthy men	Increase sperm count and motility	(33)
*Acanthopanacis senticosi*	Men with asthenospermia	Activate sperm motility	(34)
*Sesamum indicum*	Infertile men	Improve sperm count, motility and normal morphology	(35)
*Sesamum indicum*	Caffeine-induced sperm toxicity in male albino rats	Increase the weight of epididymis and sperm count, and reduce sperm head abnormalities	(36)
*Withania somnifera*	Infertile men	Improve energy metabolism and quality of semen and reproductive hormone levels	(37)
*Morinda officinalis-Lycium barbarum* with ohioensin-A, quercetin and sitosterol	Men with oligoasthenozoospermia	Reduce oxidative stress and apoptosis	(38)
*Camellia sinensis* leaves contain flavonol and epigallocatechin gallate	Wistar rats	Increase sperm concentration and viability	(39)
*Chlorella vulgaris*	Deltamethrin-intoxicated rats	Increase total sperm number and testicular antioxidant enzymes	(40)
*Chlorella vulgaris*	Rats with sodium nitrite-induced reproductive toxicity	Prevent sodium nitrite-induced alterations of sperm parameters, hormonal concentrations and testicular oxidative–antioxidant status	(41)
*Spirulina platensis*	Cadmium-intoxicated rats	Improve spermatogenesis and steroidogenesis after Cadmium exposure	(42)
*Spirulina platensis*	Furan-intoxicated rats	Improve semen quality, reproductive hormone levels and redox status in furan-intoxicated rats	(43)
*Spirulina platensis*	Mercuric chloride- intoxicated rats	Improve mercuric chloride-induced testis injuries and sperm quality alterations	(44)
*Chlorella vulgaris* and *Spirulina platensis*	Rats treated with lead acetate	Mitigate lead acetate-induced testicular oxidative stress and apoptosis	(45)
*Halopteris scoparia*	Mice with cadmium-induced reproductive toxicity	Recover sperm count, viability and motility, and reduce apoptosis	(46)
*Laminaria japonica*	Streptozotocin-nicotinamide-induced diabetic rats	Restore sperm motility and testosterone level, decrease abnormal sperm number, and inhibit lipid peroxidation	(47)
Resveratrol	Mice intoxicated with Cadmium and Lead	Improve sperm parameters, redox balance, testicular histology, and reduce signaling pathways such as Akt	(48)
Resveratrol- loaded nanostructured lipid carriers	Cryopreserved rooster sperms	Increase motility, viability, membrane function, mitochondrial activity, antioxidant capacity and reduce apoptosis	(49)
Resveratrol	Cryopreserved human sperms	Decreases DNA fragmentation. Increase markers of male fertility (protamine 1 and 2) and pregnancy success (adducin 1 alpha) by activating 5’ AMP-activated protein kinase	(50)
Curcumin	Cadmium-intoxicated mice	Increase antioxidant enzymes. Recover REDOX status	(51)
Curcumin	Artesunate-intoxicated Swiss Albino mice	Increase antioxidant enzymes. Recover REDOX status	(52)
Curcumin	Cadmium-intoxicated mice	Reduce oxidative stress via nuclear factor erythroid 2-related factor 2 (Nrf2)/antioxidant response element (ARE) pathway	(53)

REDOX, Oxidation-Reduction.

Mice fed with a high-fat diet develop a metabolic syndrome-like condition (increased body weight, hypercholesterolemia, hyperglycemia and glucose intolerance) associated with deleterious reproductive status. For instance, high-fat diet-fed mice have an increment in gonadal fat, associated with a reduction in epididymis weight and sperm count (54). However, switching from a high-fat diet to a normal diet recovers the fertility potential in obese male mice. In this sense, obese mice changing to a normal diet have a reduction in gonadal fat content, and an increment in FSH serum levels and fertility potential (55). In addition, the impact of a high-fat diet on semen parameters has been studied in a rabbit model of diet-induced hypercholesterolemia. In fact, a high-fat diet is associated with a reduction of semen volume, sperm count and motility, but an increment in sperm cholesterol content, lipid droplets, functional (acrosomal reaction) and morphological abnormalities. The testicular inefficiency is associated with reduced testosterone levels, decreased differentiation from spermatogonia to sperm cells, and increased apoptosis of germ cells. On the other hand, the addition of olive oil to the diet recovered the semen quality and sperm function dismissed by hypercholesterolemic diet in rabbits **Table 1**) (30–32).

Plants-based diets are alternative and sustainable approaches managing male infertility. Active principles and crude extracts of medicinal plants are used because of their antioxidant, anti-inflammatory, and positive effects on the testis. They have bioactive compounds such as polyphenols (anthocyanins, proanthocyanidins), phyto-oestrogens, diosgenin and thymoquinone (56). For instance, *Lepidium meyenii* (Maca), administrated to healthy men at 1.75 g/day for 3 months, increases sperm count and motility (33). Furthermore, *Acanthopanacis senticosi* activates sperm motility when administrated in humans (34). Moreover, *Sesamum indicum*, administrated at 0.5 mg/kg during 3 months, improves sperm count, motility and normal morphology (35, 36). In addition, roots of *Withania somnifera* administrated 5 g/day during 3 months improve energy metabolism and quality of semen and reproductive hormone levels in infertile men (37). *Zingiber officinale* (Ginger) powder or root are used because of their antioxidant, anti-inflammatory, anti-tumorigenic and androgenic activity. In fact, ginger contains gingerdiol, gingerol, shogaols,zingerone, zingibrene, folic acid, sesquiterpenes and vitamin C (57).

*Morinda officinalis-Lycium barbarum* coupled herbs are traditional Chinese medicines that reduce oxidative stress and apoptosis, thereby improving male fertility. These herbs contain ohioensin-A, quercetin and sitosterol that target androgen and estrogen receptors, MAPK, PI3K/Akt and glyceraldehyde-3-phosphate dehydrogenase (38). *Camellia sinensis* (green tea) leaves contain flavonol and epigallocatechin gallate and increase sperm concentration and viability when administrated for 52 days in rats (39, 57). Microalgae such as *Chlorella vulgaris* and *Spirulina platensis* improve spermatogenesis and steroidogenesis and protect against oxidative stress in rats (40–45). Algae such as *Halopteris scorapia* and *Laminaria japonica* increase sperm count, motility and testosterone levels, meanwhile decreasing sperm abnormalities, inflammation and oxidative stress, in mice and rats, respectively (46, 47, 57).

*Vitis vinífera* (grape) contains resveratrol and flavonoids (catechin, quercetin, anthocyanin and pro-anthocyanidins) with protective effects on testicles. In this sense, grape seed extracts reduce oxidative stress and apoptosis, meanwhile improve testicular histology, hormone levels, and sperm count and morphology (57). In mice, resveratrol reduces the toxic effects of cadmium and lead at the testicular level. Moreover, resveratrol prevents the development of testicular germ cell neoplasia *in situ* promoted by heavy metals. In this sense, resveratrol improves sperm parameters, redox balance, testicular histology, and reduces signaling pathways such as Akt (48). Moreover, resveratrol supplementation in a cryopreservation medium improves the post-thawed sperm quality and fertility of roosters. Indeed, rooster semen cryopreserved with 40 µM resveratrol-loaded nanostructured lipid carriers has higher motility, viability, membrane function, mitochondrial activity, antioxidant capacity and lower apoptosis than non-treated frozen sperm (49). In human cryopreserved semen samples, 15 µM resveratrol decreases DNA fragmentation mean increases markers of male fertility (protamine 1 and 2) and pregnancy success (adducin 1 alpha) by activating 5’ AMP-activated protein kinase (50).

Antioxidants lead the list of natural products that are protective agents for male infertility. In this regard, oxidative stress damages sperm membranes and DNA, thereby promoting infertility. Curcumin is a bioactive compound present in the turmeric plant *Curcuma longa* that reduce oxidative stress, lipid peroxidation and oxidative DNA damage. Curcumin increases the levels of GSH, glutathione peroxidase, superoxide dismutase and catalase. In addition, curcumin increases testosterone, FSH and LH levels in mice (51–53). Ellagic acid is a polyphenol with similar effects to curcumin. Vitamin C protects spermatogenesis, prevents sperm agglutination, and increases testosterone, FSH and LH levels. Moreover, vitamin C induces antioxidant enzymes, and reduces LDL cholesterol and lipid peroxidation. Vitamin E is an antioxidant that protects cell membrane components from oxidative damage. At reproductive level, vitamin E also protects spermatogenesis and testosterone levels (57).

### 3.3 Bioactive compounds as potential alternative treatments preventing cancer therapy-induced male infertility

Because of their availability, safety and low cost, bioactive compounds contained in fruits, vegetables and spices are potential agents for the prevention and treatment of cancer. Even though they have limitations (low bioavailability, high metabolism, poor water solubility), these molecules have antitumorigenic effects against a wide range of cancers: colon, lung, prostate, breast, gastric, liver, and brain cancer (58). Moreover, contrary to current cancer treatments, bioactive compounds are potential chemo-preventive and -therapeutic agents with low side effects on the health of patients (59, 60).

Bioactive compounds target cancer cells, macrophages and adipocytes in the tumor microenvironment, thereby preventing cancer development and progression (61). In this way, curcumin, myricetin, geraniin, genistein, tocotrienol, fucoxanthin, anthocyanin, epigallocatechin gallate, gallic and ellagic acids have anti-proliferative, pro-apoptotic and anti-metastatic effects. For instance, curcumin, in nanoparticles, piperine, phospholipid complexes and liposomes, inhibits PI3K/Akt and NF-κB pathways, but upregulates p53 and Bax expression, thereby promoting apoptosis of cancer cells. In addition, curcumin downregulates MMP-9 expression and reduce the metastatic potential of cancer cells (59). Furthermore, *Tripterygium wilfordii* used in the Chinese medicine contains bioactive compounds such as triptolide, celastrol and tripchlorolide that also inhibit PI3K/Akt and NF-κB pathways thereby exerting anticancer and anti-inflammatory effects (62).

As previously mentioned, several plant-derived bioactive compounds are effective in improving male infertility. In addition, these compounds have been shown to reverse cancer-induced infertility. **Table 2** summarizes the sources and bioactive compounds known to prevent cancer-associated male infertility by controlling the detrimental effects of cancer therapy.

**Table 2:** Bioactive compounds preventing cancer therapy-induced male infertility.

Source or Bioactive compound	Model	Chemotherapy	Dose	Effect	Reference
Ginger extract	Rat	5 mg/kg busulfan, IP	100-150 mg/kg for 48 days	↑ volume of seminiferous tubules ↑ sperm count ↑ testosterone levels	(63)
Ginger extract	Rat	100 mg/kg cyclophosphamide, IP	300-600 mg/kg, oral	↑ antioxidant and testosterone serum levels	(64)
Fucoxanthin-rich extract obtained from *Sargassum glaucescens*	Hamster	7 mg/kg cisplatin, IP	100-500 mg/kg, oral	↑ antioxidant enzymes ↑ testosterone serum levels ↑ sperm count and motility ↓ sperm abnormality	(65)
Curcumin	Rat	5 mg/kg cisplatin, IP	100 mg/kg/day for 7 days	↓ NF-κB and caspase-3 activation	(66)
Curcumin	Rat	7 mg/kg cisplatin, IP	200 mg/kg/day for 7 days	↓ MAPK and NF-κB	(67)
Curcumin nanocrystals	Mouse	200 mg/kg cyclophosphamide, IP	4 mg/kg	↑ sperm activity ↑ sperm chromatin condensation ↑ seminiferous tubule architecture ↓ germ cells apoptosis	(68)
Ellagic acid	Mouse	5 mg/kg cisplatin, IP	10 mg/kg for 5 weeks, oral	↑ sperm count	(69)
Vitamin E and L-carnitine	Rat	20 mg/kg methotrexate, IP	250 mg/kg vitamin E with 500 mg/kg L-carnitine	↓ malondialdehyde ↑ superoxide dismutase	(70)
Resveratrol	Rabbit	5 mg/kg paclitaxel, IV	4 mg/kg for 8 weeks, IV	↓ DNA fragmentation and abnormal DNA integrity in epididymal sperms	(71)
Resveratrol	Mouse	30 mg/kg busulfan, gavage	100 mg/kg/day for 2 weeks	↑ proliferation of germ cells	(72)

IP, Intraperitoneal; IV, Intravenous.

For instance, ginger and algae extracts have beneficial reproductive effects post cancer-therapy (57). Rats treated with a single intraperitoneal (IP) injection of 5mg/kg busulfan (chronic myelogenous leukemia treatment) and with 100-150 mg/kg ginger extract for 48 days have increased volume of seminiferous tubules, sperm count and testosterone levels, previously impair by busulfan (**Table 2**) (63). Another group demonstrated that oral administration of 300-600 mg/kg ginger extract recovers the epithelium thickness and germ cell count of rat testis affected after a single IP dose of 100 mg/kg cyclophosphamide treatment by increasing antioxidant and testosterone serum levels (64). In addition, oral administration of fucoxanthin-rich brown algae *Sargassum glaucescens* extract ameliorates cisplatin-induced testicular damage in hamsters. Hamsters were intraperitoneal injected with 7 mg/kg cisplatin and treated with 100, 200 and 500 mg/kg fucoxanthin extract orally administrated. In fact, fucoxanthin-enriched extract recovers the testosterone level, seminiferous tubules morphology and sperm number, motility and morphology affected by cisplatin treatment (65).

Additionally, several bioactive compounds such as curcumin, ellagic acid and vitamin E are involved in reverting cancer-induced male infertility (57). For instance, rats treated with a single IP dose of 5 mg/kg cisplatin and 100 mg/kg/day curcumin during 7 days reverse testicular damage by reducing NF-κB and caspase-3 activated by cisplatin (66). Rats treated with a single IP dose of 7 mg/kg cisplatin and orally administrated 200 mg/kg/day curcumin for 10 days recover oxidative stress and testicular damage induced by cisplatin via mitogen-activated protein kinase and nuclear factor-kappa B signaling pathways (67). Mice treated with 4 mg/kg curcumin nanocrystals recover the negative effects of cyclophosphamide in sperm activity, sperm chromatin condensation, and seminiferous tubule architecture, meanwhile have a reduction in germ cells apoptosis induced by the treatment with 200 mg/kg cyclophosphamide (IP) (68). Mice with colon cancer treated with 5 mg/kg cisplatin (IP) have a reduction in sperm count which is recovered after 5 weeks of treatment with 10 mg/kg ellagic acid (oral) (69). Rats receiving 250 mg/kg vitamin E with 500 mg/kg L-carnitine control the oxidative stress induced by the treatment with 20 mg/kg methotrexate (IP) via reducing malondialdehyde and increasing superoxide dismutase (70). In rabbits, the treatment with 4 mg/kg resveratrol (intravenous) for 8 weeks ameliorates the DNA fragmentation and abnormal DNA integrity in epididymal sperms induced by 5 mg/kg paclitaxel (71). In addition, 20 µM resveratrol induces the proliferation of spermatogonia stem cells *in vitro*. In mice, 100 mg/kg/day resveratrol for 2 weeks promotes the proliferation of germ cells thereby reversing the loss of spermatogenic cells in the testis and sperm cells in the epididymis induced by 30 mg/kg busulfan treatment (gavage) (72).

## 4 Conclusion

**Figure 1:**
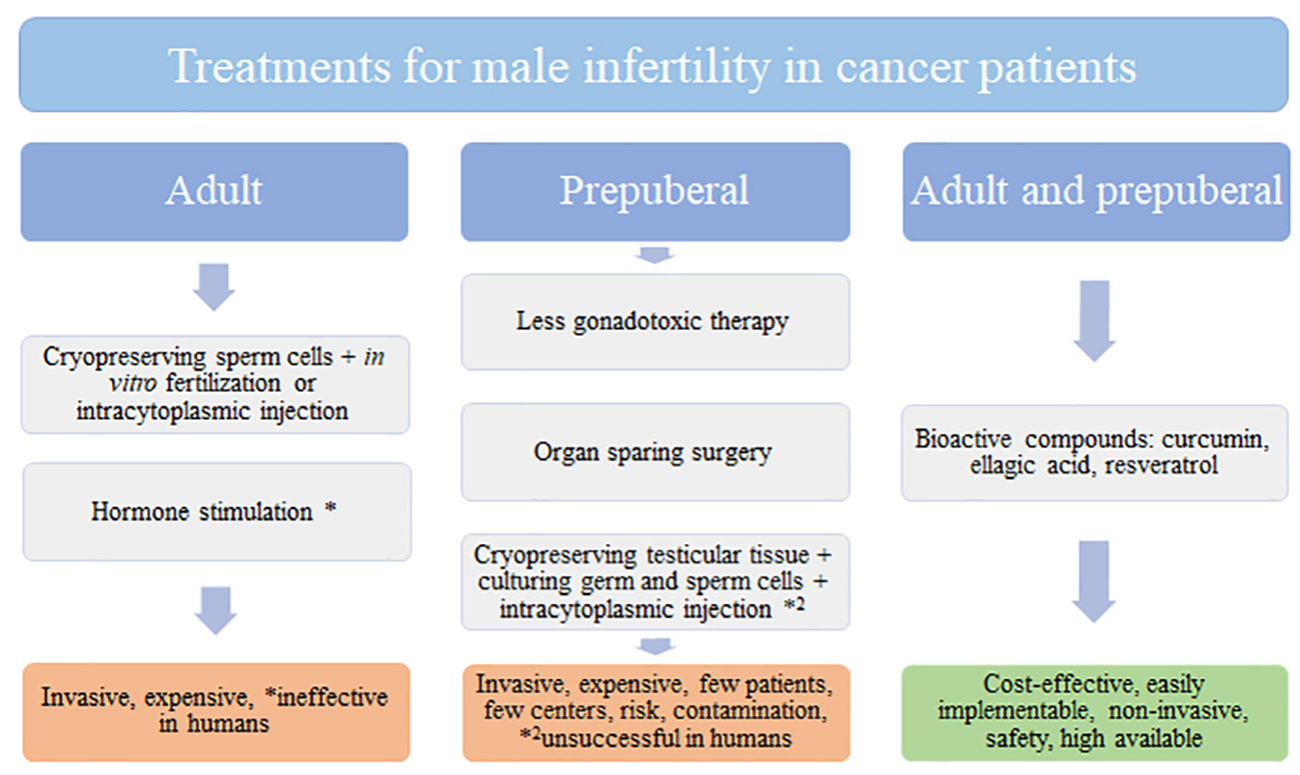
Treatments for male infertility in cancer patients.

Cancer incidence is increasing worldwide and men are more affected than women. Current cancer treatments involve surgery, chemotherapy and radiation, which have side-effects such as infertility. Moreover, male infertility is responsible for 50% of couples with fertility problems. Adult cancer patients are subjected to invasive and expensive techniques to recover fertility after cancer treatment such as cryopreservation of sperm cells and *in vitro* fertilization (**Figure 1**). However, prepuberal patients without sperm cells are unable to access to these techniques. In this age group, cryopreserving testicular tissue to culture germ and sperm cells and do intracytoplasmic injection seems to be another invasive, expensive technique that is unsuccessful in humans. In this way, looking for alternative treatments for cancer and male infertility is a need to alleviate patient suffering post-cancer survival. Natural products rich in bioactive compounds are increasing interest in this scenario as alternative, novel, non-invasive agents to prevent and treat cancer preserving male fertility. Curcumin, ellagic acid, and resveratrol seem to be potential compounds that recover testicular function by increasing proliferation, reducing oxidative stress, inflammation, and apoptosis of germ cells. However, further research regarding bioavailability, solubility and metabolism of these natural compounds must be taken in consideration to improve the current therapy approaches.

## Author contributions

LS: Conceptualization, Funding acquisition, Writing – original draft, Writing – review & editing. MM-C: Funding acquisition, Writing – review & editing.
